# Network analysis of hyphae forming proteins in *Candida albicans* identifies important proteins responsible for pathovirulence in the organism

**DOI:** 10.1016/j.heliyon.2019.e01916

**Published:** 2019-06-13

**Authors:** Sanjib Das, Rajabrata Bhuyan, Angshuman Bagchi, Tanima Saha

**Affiliations:** a Department of Molecular Biology & Biotechnology, University of Kalyani, West Bengal, 741235, India; b Department of Biochemistry & Biophysics, University of Kalyani, West Bengal, 741235, India

**Keywords:** Bioinformatics, Microbiology

## Abstract

*Candida albicans* causes two types of major infections in humans: superficial infections, such as skin and mucosal infection, and life-threatening systemic infections, like airway and catheter-related blood stream infections. It is a polymorphic fungus with two distinct forms (yeast and hyphal) and the morphological plasticity is strongly associated with many disease causing proteins. In this study, 137 hyphae associated proteins from *Candida albicans* (*C. albicans)* were collected from different sources to create a Protein-Protein Interaction (PPI) network. Out of these, we identified 18 hub proteins (Hog1, Hsp90, Cyr1, Cdc28, Pkc1, Cla4, Cdc42, Tpk1, Act1, Pbs2, Bem1, Tpk2, Ras1, Cdc24, Rim101, Cdc11, Cdc10 and Cln3) that were the most important ones in hyphae development. Ontology and functional enrichment analysis of these proteins could categorize these hyphae associated proteins into groups like signal transduction, kinase activity, biofilm formation, filamentous growth, MAPK signaling etc. Functional annotation analysis of these proteins showed that the protein kinase activity to be essential for hyphae formation in *Candida*. Additionally, most of the proteins from the network were predicted to be localized on cell surface or periphery, suggesting them as the main protagonists in inducing infections within the host. The complex hyphae formation phenomenon of *C. albicans* is an attractive target for exploitation to develop new antifungals and anti-virulence strategies to combat *C. albicans* infections. We further tried to characterize few of the most crucial proteins, especially the kinases by their sequence and structural prospects. Therefore, through this article an attempt to understand the hyphae forming protein network analysis has been made to unravel and elucidate the complex pathogenesis processes with the principal aim of systems biological research involving novel Bioinformatics strategies to combat fungal infections.

## 1 Introduction

*Candida albicans* is a pathogenic fungus belonging to the family Saccharomycetaceae which causes life-threatening infections in humans with mortality rate of 40–60% [1, 2, 3]. It is an opportunistic pathogen causing circumscribed infections of the skin, nails, and mucocutaneous membranes in healthy people, whereas, becomes aggressive in immune deficient patients due to malignancy, inherited disease, concurrent infection, or medical intervention [4, 5, 6, 7, 8]. Among *Candida* spp., *C*. *albicans*, the main pathogen in this genus, is responsible for the majority of all forms of candidiasis [9]. In nosocomial urinary tract infections, approximately 80% is caused by *C. albicans* [10, 11]. Indeed, in the United States, the fourth most common hospital borne systemic infections are caused by *Candida* sp. with crude mortality rates of up to 50% [12, 13]. Approximately 75% of women are prone to get infected from vulvovaginal candidiasis (VVC) at least once in their lifespan with 40–50% chance of additional episode [14, 15]. Furthermore, 5–8% amongst them suffer from at least four recurrent VVC in a year [16].

*C. albicans*is a polymorphic fungus that can grow either as ovoid-shaped budding yeast (blastopore), as elongated ellipsoid cells with compressions at the septa (pseudohyphae) or as parallel walled true hyphae [17]. The yeast form is believed to be primarily involve in dissemination, whereas, hyphal form shows more invasiveness [18, 19]. *Candida* species infect the host by these significant virulent morphological structures-pseudohyphae, (e.g., *Candida tropicalis*, *Candida parapsilosis*, *Candida guilliermondii*, and *Candida lusitaniae)* [20, 21] and hyphae (*C. albicans*, *C. dubliniensis*, and *C. tropicalis*) [17]*.* The genome sequences of different *Candida* species have indicated that many of the genes involved in yeast to filamentous transition are evolutionarily conserved [22]. It is significantly noted that the approximately 85% of identified filamentous genes of *C. albicans* are homolog to other *Candida* species [23]. *C. albicans* shows greater expansion in the number of genes relative to most of the other *Candida* species belonging to the same family. Consistent with this thought, less pathogenic *Candida* species have reduced the ability to produce virulent factors that are required for adhesion and invasion in the host cell in comparison to pathogenic hyphae forming *C. albicans* [24]. The hyphae formation is an important part in the infection process of *C. albicans,* as it helps by promoting tissue penetration in host epithelial and endothelial cells and also avoids host immune system [25, 26]. *C. albicans* shows morphological plasticity and the transition from yeast to filamentous form in the host is a critical virulence determinant of infections [27]. The hyphal morphogenesis has always been associated with virulent proteins that govern simultaneously in a co-regulated fashion both virulence and hyphal growth [28, 29, 30]. Therefore, it is necessary to understand the mechanisms of hyphae formation in *C. albicans* and the role of virulent proteins to elucidate the complex pathogenesis processes.

In this work, we have implemented several bioinformatics and systems biology approach combining text mining methods to analyze and interpret the Protein-Protein Interaction (PPI) network of proteins that are involved in *C. albicans* phenotypic plasticity by conversion from yeast to hyphae formation and development. We used *Saccharomyces cerevisiae*, a fungal model organism as a control for the validation of interactions and different aspects related to *Candida* hyphae forming proteins. With the limitation of antifungal drug availability and enhancing populations of susceptible patients, it is essential to understand the mechanisms of hyphae formation in order to develop new strategies for treating candidiasis. We have tried to infer the essential proteins and their regulation through the network analysis, which will be highly beneficial to understand the resistance mechanisms as well as for further development of anti-fungal therapy.

## 2 Materials and methods

### 2.1 Dataset preparations and validations

Proteins that are known to participate in hyphae formations in *C. albicans* were collected from literature and Candida Genome Database (CGD). The CGD is a well-organized repository containing various kinds of genomic, proteomic, morphology and annotation related information from four Candida species including *C. albicans* [31]. The proteomic data of *C. albicans* revealed that more than 70% of enlisted proteins are uncharacterized. Orthology analyses of these proteins were carried out by using NCBI BLAST [32] with other *Candida* species and *Saccharomyces cerevisiae*. We used *Saccharomyces cerevisiae*, a fungal model organism, as control for the validation and analyses of different aspects of protein-protein interactions (PPI) amongst the hyphae forming *Candida* proteins. All the PPI data were retrieved from several online resources (eg. STRINGS, BIOGRID, CGD & UNIPROT) and literatures [33, 34, 35]. The STRINGS and BIOGRID are known for storing information about both physically and functionally interacting proteins, whereas the CGD & UNIPROT contain information of interactions validated by experimental findings. Following the aforementioned databases, we built a full interaction dataset containing the details of the proteins that have well-defined roles in hyphae developments using an in-house pipeline written in Perl. The final datasets consisted of a total of 137 proteins and 714 unique interactions. On the basis of their roles, the proteins were classified as either promoting hyphae formation or suppressing the process. The interactions were also marked as either physical or functional.

### 2.2 Building and visualizing the network

The total PPI datasets were arranged in a network form by Cytoscape 3.6.1 [36]. The Cytoscape defines PPI networks as graphs in terms of nodes and edges, which represent the proteins and their associated interactions, respectively. All the edges are considered as ‘unidirectional’ in the network, and duplicate edges including ‘self-loops’ were removed. The same Cytoscape was further used for visualization and analyzing the network using its diverse plugins integrated for multiple functions. Independent colour codes were used for distinguishing the nodes for their functions and topological attributes.

### 2.3 Network topology analysis

Topological analyses were performed by employing “Network Analyzer” plugin of Cytoscape [37]. The quality of network architecture can be validated by different topological attributes such as degree *k*, clustering coefficient *C(n)*, Betweenness centrality *BC(n)* and Closeness centrality *CC(n)*. Degree *k* defines the number of directly connected neighbors of a node. The clustering coefficient *C(n)* is a measure of the degree of a node that has a greater probability to cluster together in the network. Similarly, Betweenness centrality *BC(n)*is the relative frequency of all paired shortest paths of a particular node in a network gives the information about the extent of interactions that a node mediates in a network. Closeness centrality *CC(n)* shows the spreading of information of a node and is defined as the reciprocal of the average shortest path length in a network [38, 39, 40, 41, 42, 43]. *CC(n)* would represent the strength of the interactions. A shorter path length would mean a stronger interaction. Another important attribute is the node degree distribution *P(k)*, which aids to declare whether a network is random or scale-free, and is calculated by fitting the power law using equation $y=ax^{b}$; where ‘a’ is a constant and ‘b’ is denoted as an exponent. In this study, power law of *P(k)* has been used to evaluate the robustness of the network [38].

### 2.4 Identification of hub proteins

Hub proteins are the ones which have the maximum number of interacting partners in a network [43]. Here, the proteins containing more than 20 interactions were considered as Hub proteins, estimated from topological parameter degree *k*. Additionally, the Cytohubba plugin of Cytoscape was used to identify the hub proteins/nodes from the network [44]. There are a total of 11 different methods implemented in Cytohubba to analyze the network feature to rank the nodes accordingly.

### 2.5 Modular analysis and sub-network generation

Molecular complex detection (MCODE) plugin of Cytoscape was used to identify highly connected local sub-networks from the total PPI network [45]. Module identification is based on the principle of two interacting proteins having high probabilities of interactions with each other. The MCODE algorithm generates the modular clusters from PPI network through vertex weighting using local neighborhood density and outward traversal from dense protein node to discover dense regions. The parameters set for modular analysis were of degree cutoff = 2, haircut = true, node score cutoff = 0.2, k-score = 2, and maximum depth = 100. Top five clusters were considered further on the basis of MCODE score ≥4.

### 2.6 Functional enrichment and ontology analysis

Functional annotations of top scoring clusters were performed online at DAVID Bioinformatics Resources server [46]. Overall annotation analyses of the whole PPI network were executed using ClueGO plugin of Cytoscape [47]. ClueGO is known for integrating Gene Ontology (GO) terms as well as KEGG/BioCarta pathway terms and generates functionally organized networks on the basis of their annotations. Several gene ontology (GO) terms such as biological processes, molecular function and cellular components for *C. albicans* were retrieved and subjected to ClueGO analyses. Finally, two-sided hypergeometric test (enrichment/depletion), with Bonferroni steps down for *pV* correction at 0.05 significance level (*p*-value) and kappa score of 0.4 were set as threshold to analyze the network.

### 2.7 Structure modeling of candida kinase domain

Amino acid sequence of Candida Chk1 kinase domain (AA, 358–637) was downloaded from Uniprot (ID: Q5AHA0), and subjected to the template-based threading and modeling server I-TASSER [48]. The best modeled structure generated by I-TASSER was further refined by Smart Minimizer of Discovery Studio (DS) 2.5 with RMS gradient of 0.1, consequently, its stereochemistry was checked through SAVES server http://services.mbi.ucla.edu/SAVES.

## 3 Results

### 3.1 Construction of network

**Fig. 1:**
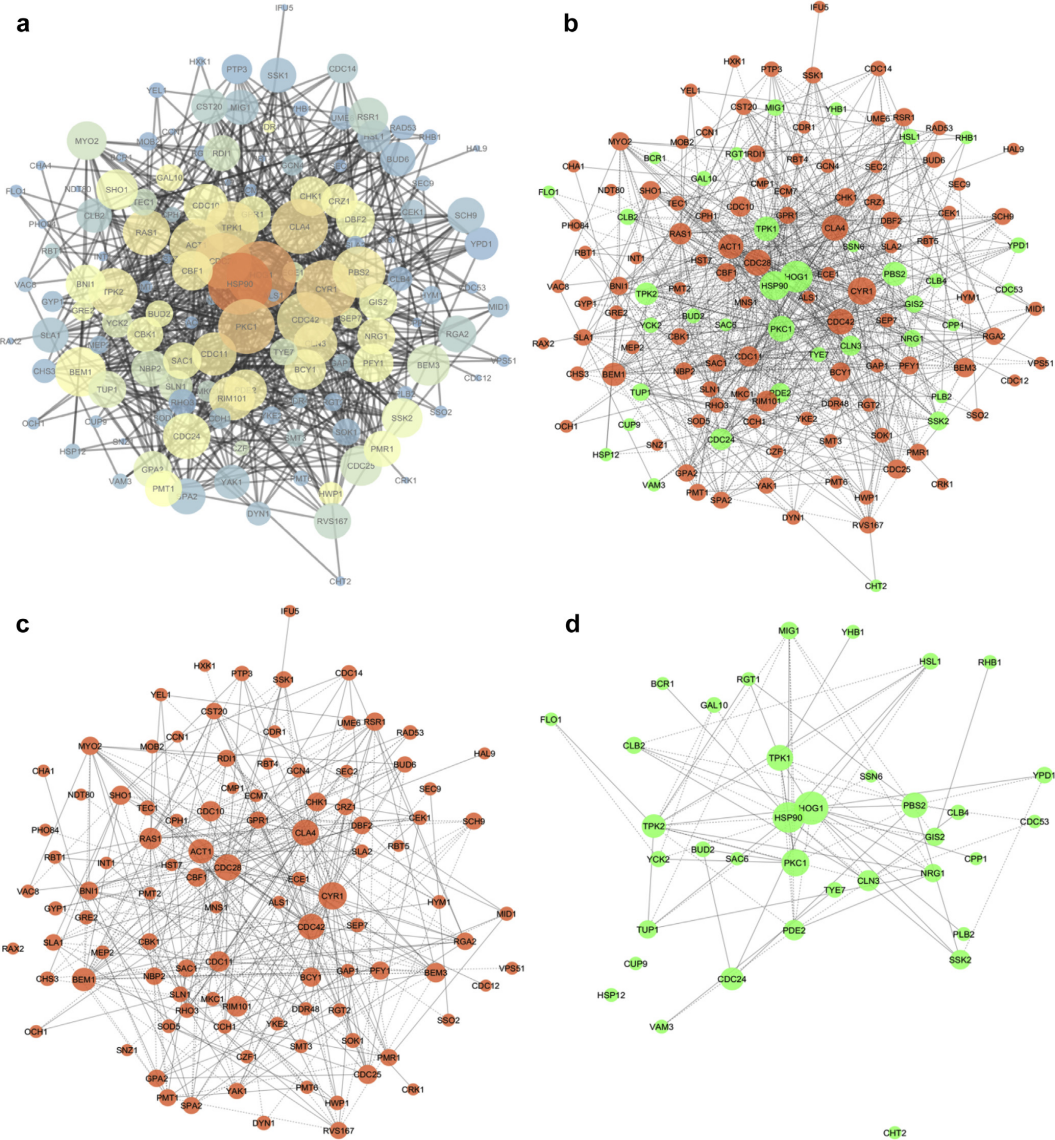
General representation of the network; (a) Node sizes were set on their degrees. The nodes were colored by their betweenness centrality values (red to grey). (b) Node sizes were set on their degrees. The nodes were colored by their roles in hyphae formation and development (red-promoting; green-suppressing). (c) Interactions among the hyphae promoting nodes. (d) Interactions among the hyphae suppressing nodes.

PPI network analysis is a crucial approach towards the understanding of the mechanisms of complex biological reactions and their possible outcomes. In the present work, we focused on building comprehensive network of proteins to analyze their modes of interactions leading to hyphae formation in *C. albicans*. For this, we collected the information of a total of 137 potent proteins having association with hyphae formation and regulation from published literatures [49, 50, 51, 52, 53] and other online resources [Candida Genome Database; Saccharomyces Genome Database; STRING; BioGRID; UniProt; PubMed; DAVID]. We used the aforementioned collected information to analyze the modes of interactions (i.e. physical or genetic) and integrated them within the network through Cytoscape. Finally, the PPI network was constructed with experimentally validated interactions consisting of 137 nodes and 714 edges. Among the 137 proteins, 101 proteins were identified as promoting hyphal growth, whereas 36 were supposed to be suppressing the hyphae formations. General features of the network were presented in Fig. 1.

### 3.2 Topology analysis

**Fig. 2:**
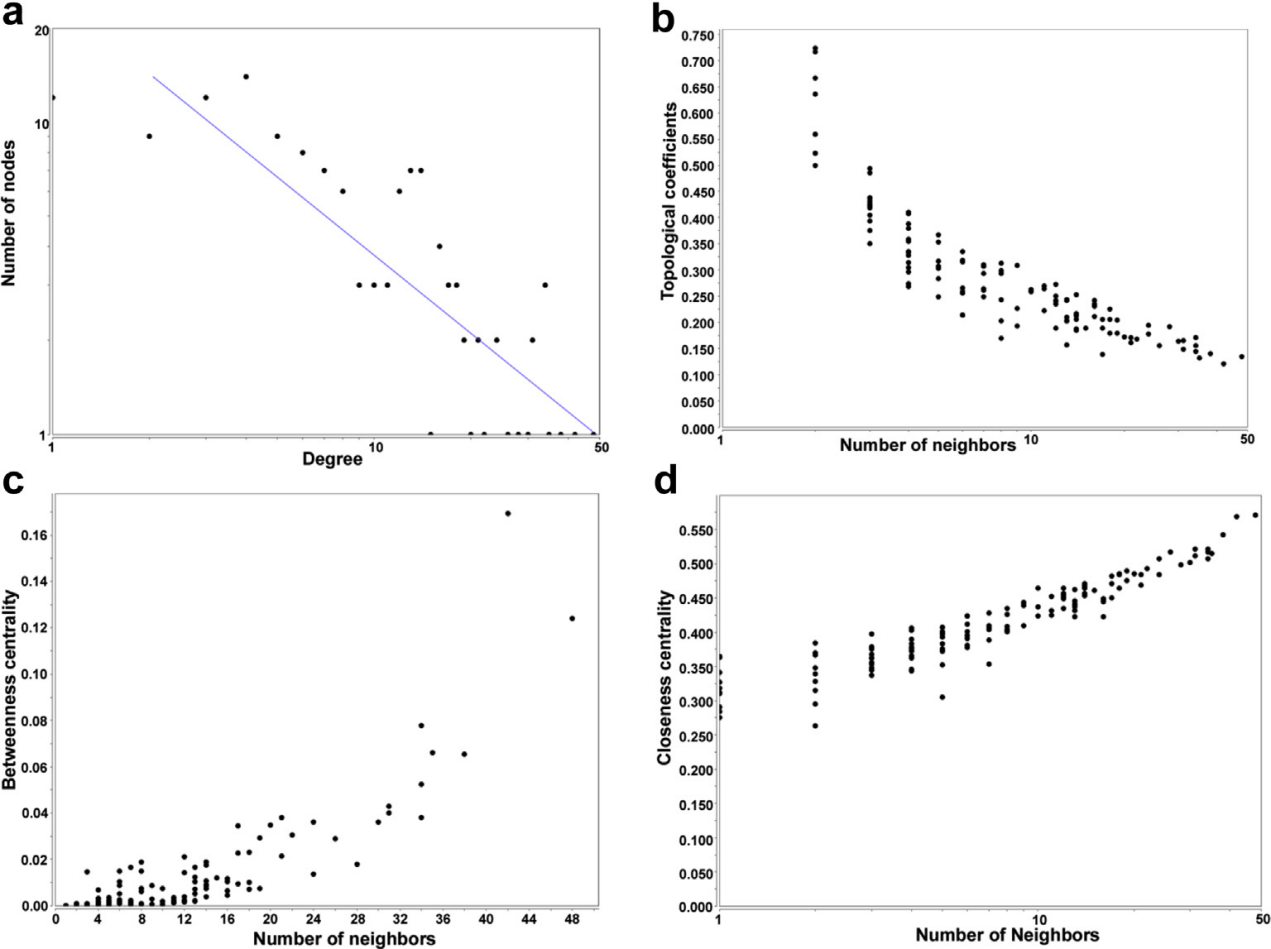
Topological attributes of the network; (a) Node degree distribution of the network with power fitted. (b) Distribution of topological coefficients. (c) Betweenness centrality. (d) Closeness centrality.

PPI networks or biological networks show distinctive topological characteristics, which make them different from other random networks (Fig. 2). The most important feature is the power law of node degree distribution which gives information about the robustness of the network [43]. It has been stated that the exponent form of the power law in any scale free biological network should be less than 2 [38,43]. In our case, the exponent ‘*b’* was found to be -0.833, which signifies its reliability and the importance of hubs in the network (Fig. 2a).

Other various parameters of the PPI networks, such as clustering coefficient *C(n)*, network centralization, and network density were found to be 0.325, 0.280, and 0.077, respectively. The maximum value of average clustering coefficient (0.325) was observed to justify the network with impressive measurement of nodes to be clustered together. Similarly, the network centralization score signified the importance of each node with good resemblance to the ratio of actual connections to the total possible connections within a network (density). The number of shortest paths was 18632 in the *Candida* hyphae PPI network, which would indicate that the nature of connectivity of the proteins was relatively high. The result also reveal that the transmission of biological information in the network was achieved through only a few steps as these proteins were involved in hyphae formation in the species by responding to various physiological and environmental clues. Similarly, the value of the degree centrality could identify the important nodes in the network on the basis of number of interactions, which were distinguished in terms of shared pathways or biological processes. The distribution of closeness centrality *CC(n)*, and Betweenness centrality *BC(n)* were presented in Fig.2c and 2d. *CC(n)* of a node in a network gives the idea about information that is passed from one node to another by measuring the number of shortest paths passing through the nodes from a PPI network [38]. Here Hog1 had the highest value of 0.57142857. Likewise, the *BC(n)* analysis of nodes revealed the proteins that could act as bridges or connect distant proteins together in the network.

### 3.3 Hub protein analysis

**Fig. 3:**
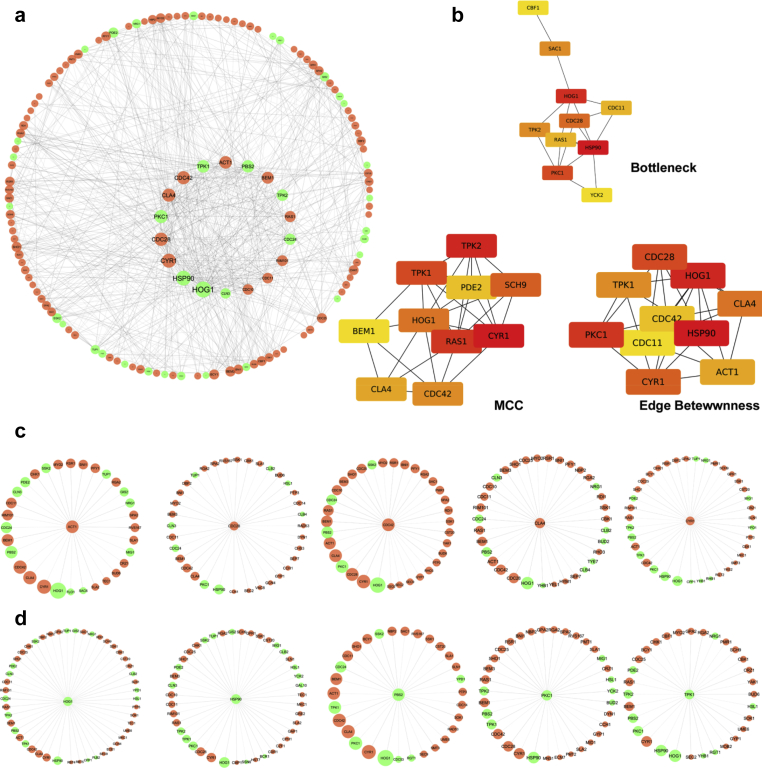
Representation of hub proteins. (a) Circle view of the whole network identifying the nodes with more than 20°. (b) Hub proteins identified by CytoHubba using different algorithms. (c) Individual interactions of top five hyphae promoting proteins. (d) Individual interactions of top five hyphae suppressing proteins.

Both the network centrality as well as CytoHubba plugin was used to identify the hub proteins throughout the network. In this network, the Hsp90 and Hog1 proteins exhibited the highest *BC(n)* presuming these two to act as bridges, or bottlenecks, and were necessarily responsible for keeping the other nodes of the network intact (Fig. 3a). Top ten hub proteins identified by Bottleneck, MCC and Edge betweenness algorithms of CytoHubba were presented in Fig. 3b. As observed from other PPI networks, a node degree of less than 20 maybe considered to have not so important roles in the said biological process (i.e. not solely performing) [54]. Those were non-seed proteins and were not considered as hubs. In our study the top five hub proteins each from promoting and suppressing groups were presented in Fig.3c and d. From the above analysis, it could possibly be stated that the following 18 proteins, viz., Hog1, Hsp90, Cyr1, Cdc28, Pkc1, Cla4, Cdc42, Tpk1, Act1, Pbs2, Bem1, Tpk2, Ras1, Cdc24, Rim101, Cdc11, Cdc10 & Cln3 were the proteins that might have the highest degrees, betweenness and closeness centrality values and could act as hubs or bottlenecks in the PPI network, among which 10 were suggested to promote and 8 to suppress the hyphae formation (Table 1). In a cellular system, it has been proposed that most interacting networks follow the overall broad-scale topology, where less number of proteins is regarded as hubs and most proteins interact with fewer partners [55]. The current network would represent only the interconnection among the proteins that are involved in hyphae formation, which is just a part of whole interactomes. However, such investigations might pave the first step towards the understanding of hyphae forming mechanisms in *C. albicans* from a systems biology point of view.

**Table 1:** Topological attributes for hub proteins.

Name	Degree	Betweenness Centrality *BC(n)*	Closeness Centrality *CC(n)*	Bottleneck	MCC	Edge Betweenness
Hog1	*48*	*0.12396158*	*0.57142857*	*14*	*26625*	*2275.935*
Hsp90	*42*	*0.16951442*	*0.56903766*	*14*	*12597*	*3112.285*
Cyr1	38	0.0654404	0.54183267	10	31090	1201.486
Cdc28	35	0.0660763	0.51515152	7	6064	1213.161
Pkc1	*34*	*0.07775958*	*0.5210728*	*6*	*2221*	*1427.666*
Cla4	34	0.05254553	0.51711027	13	16936	964.736
Cdc42	34	0.03810069	0.50746269	14	18462	699.5287
Tpk1	*31*	*0.04288084*	*0.5210728*	*5*	*27301*	*787.2923*
Act1	31	0.04014922	0.5112782	19	4035	737.1397
Pbs2	*30*	*0.03608228*	*0.50184502*	*4*	*4674*	*662.4706*
Bem1	28	0.01798415	0.4981685	3	16672	330.189
Tpk2	*26*	*0.02883792*	*0.51711027*	*3*	*29211*	*529.4643*
Ras1	24	0.03621418	0.50746269	2	29045	664.8924
Cdc24	*24*	*0.01369953*	*0.48398577*	*3*	*15754*	*251.5233*
Rim101	22	0.03041436	0.49275362	4	813	558.4076
Cdc11	21	0.0380035	0.48398577	2	372	697.7443
Cdc10	21	0.02138152	0.46896552	2	302	392.5646
Cln3	*20*	*0.03490596*	*0.48571429*	*3*	*159*	*640.8734*

Proteins that participates in suppressing hyphae were mentioned in italics.

*BC(n)* value indicates the extent of interactions that a node mediates in a network.

*CC(n)* represents the degree of a node that has a greater probability to cluster together.

Bottleneck, MCC, Edge Betweenness scores are the output of three algorithms used by CytoHubba plugins to generate the hub proteins.

### 3.4 Sub-network and enrichment analysis

**Fig. 4:**
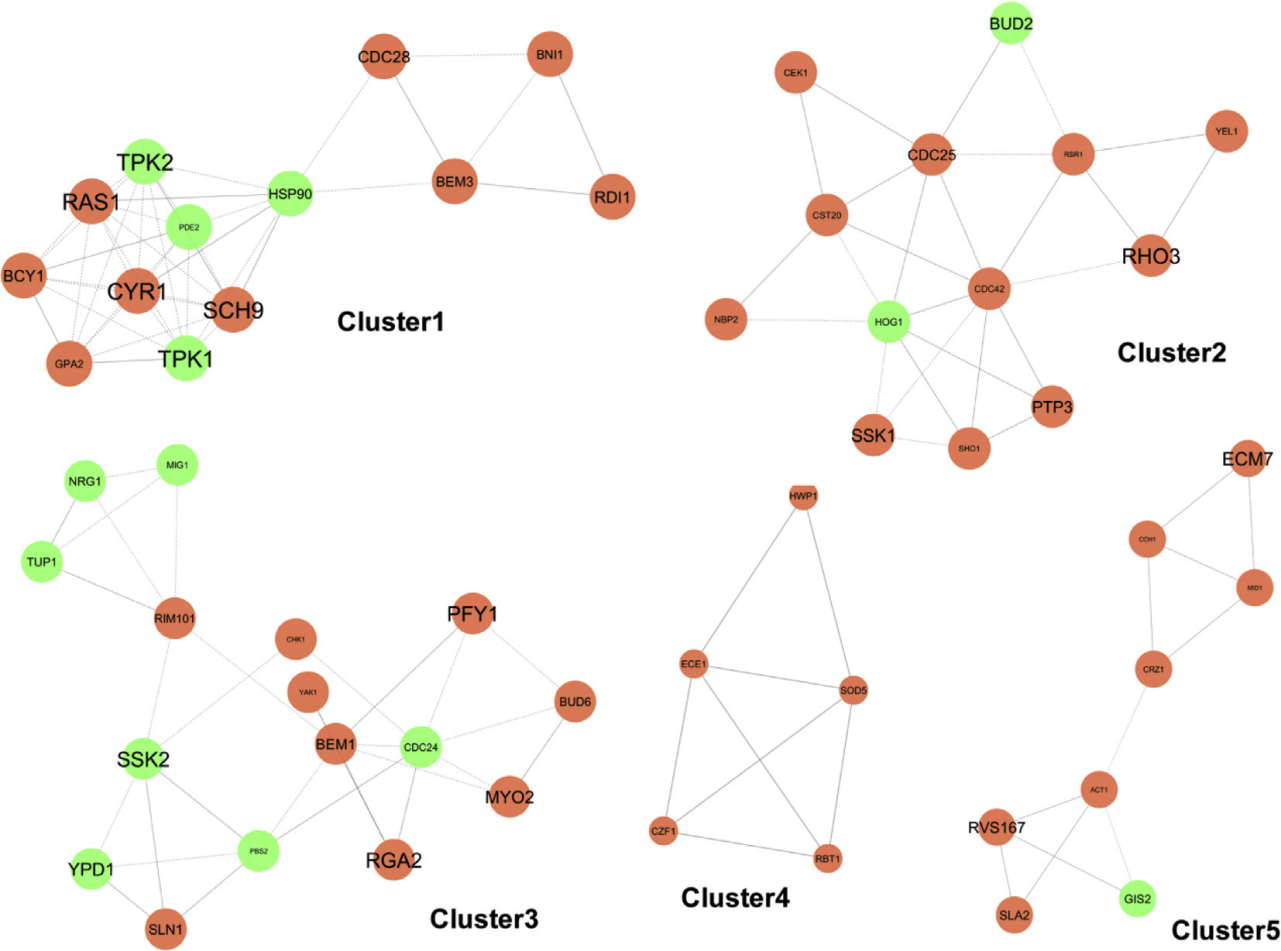
Sub-networks generated by MCODE.

The sub-networks generated by MCODE plugin were ranked on the basis of their confidence score, which is an indicator of their likeliness to form real protein complexes [45]. Out of five clusters detected by MCODE, three clusters (MCODE score: 6.833, 4.333 & 4) were selected for enrichment analysis. The first two modules contained 13 nodes of each and edges of 41 and 26, respectively; whereas the third one had 16 nodes and 30 edges (Fig. 4). All the three modules were found to be associated with many statistically significant GO terms. The proteins present in cluster 1 were found to belong to the following classes: Nucleotide-binding (*P* value: 3.1E-10), Serine/threonine-protein kinase (*P* value: 3.2E-7), Cellular response to starvation (*P* value: 3.4E-7), Filamentous growth (*P* value: 1.3E-6) and cAMP-mediated signaling (*P* value: 3.8E-6).

On the other hand, the proteins in cluster 2 were annotated with versatile functions such as MAPK signaling pathway - yeast (*P* value: 1.3E-16), small GTPase mediated signal transduction (*P* value: 4.7E-11), filamentous growth (*P* value: 2.1E-9), small GTPase superfamily (*P* value: 1.4E-8), nucleotide-binding (P value: 4.2E-8) and fungal-type cell wall organization (*P* value: 8.1E-8). Similarly, the cluster 3 was detected as the largest one and associated with MAPK signaling pathway (*P* value: 6.7E-15), two-component regulatory system (*P* value: 2.3E-11), kinase (*P* value: 6.4E-8), signal transduction histidine kinase (P value: 7.6E-7), phosphoprotein (*P* value: 1.4E-6), and cellular response to farnesol (*P* value: 2.0E-6). Total 12 previously identified hub proteins were rediscovered in these clusters, where Cyr1, Ras1, Tpk2, Tpk1, Cdc28, Hsp90 were detected in cluster 1, Hog1 & Cdc42 in cluster 2, and Pbs2, Cdc24, Rim101 & Bem1 in cluster 3.

### 3.5 Classification of interactions on the basis of enrichment analysis

The ClueGO plugin of Cytoscape was used to create the network of over-represented nodes based on predefined kappa score level. It generates a dynamical network structure from a gene list of interest and projects functionally grouped terms by means of kappa statistics to link the attributes in the network [56]. The ontology and pathway enrichment analysis of the whole set of proteins produced three different functional characterization terms such as biological process, molecular function and cellular components.

**Fig. 5:**
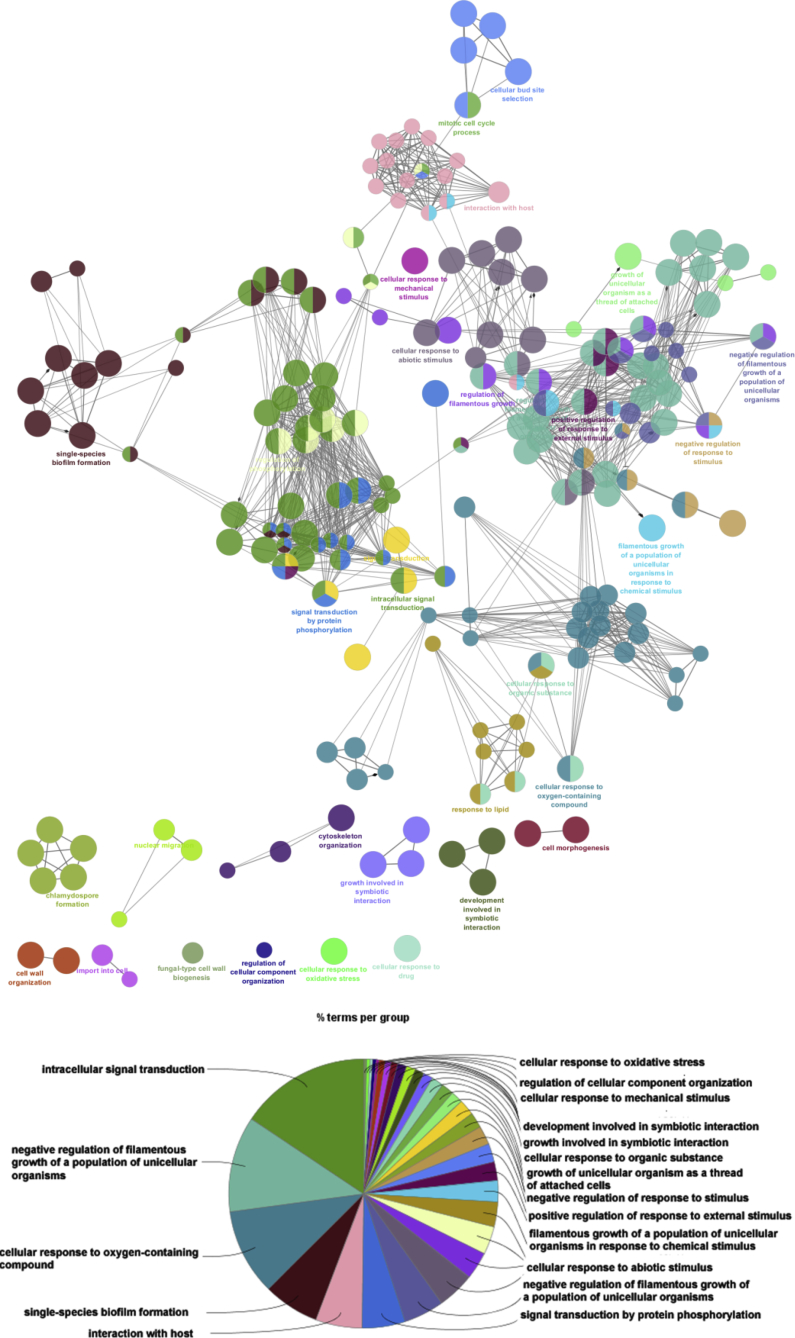
ClueGO analysis of top scoring clusters from biological process.

Annotations of the proteins against 6971 reference gene sets were functionally grouped in important biological process such as intracellular signal transduction (GO:0035556), regulation of filamentous growth of a population of unicellular organisms (GO:1900428), cellular response to oxygen-containing compound (GO:1901701), single-species biofilm formation (GO:0044010), interaction with host (GO:0051701), signal transduction by protein phosphorylation (GO:0023014), negative regulation of filamentous growth of a population of unicellular organisms (GO:1900429), cellular response to abiotic stimulus (GO:0071214), filamentous growth of a population of unicellular organisms in response to chemical stimulus (GO:0036171), positive regulation of response to external stimulus (GO:0032103) (Fig. 5 and supplementary table Table S1). In total, 130 proteins were clustered in any category of biological process. Out of 137, seven proteins like Tsp1, Ydr174, Yel1, Yer67, Yer73, Ylr63 and Ymr90 could not be grouped and remained un-annotated.

**Fig. 6:**
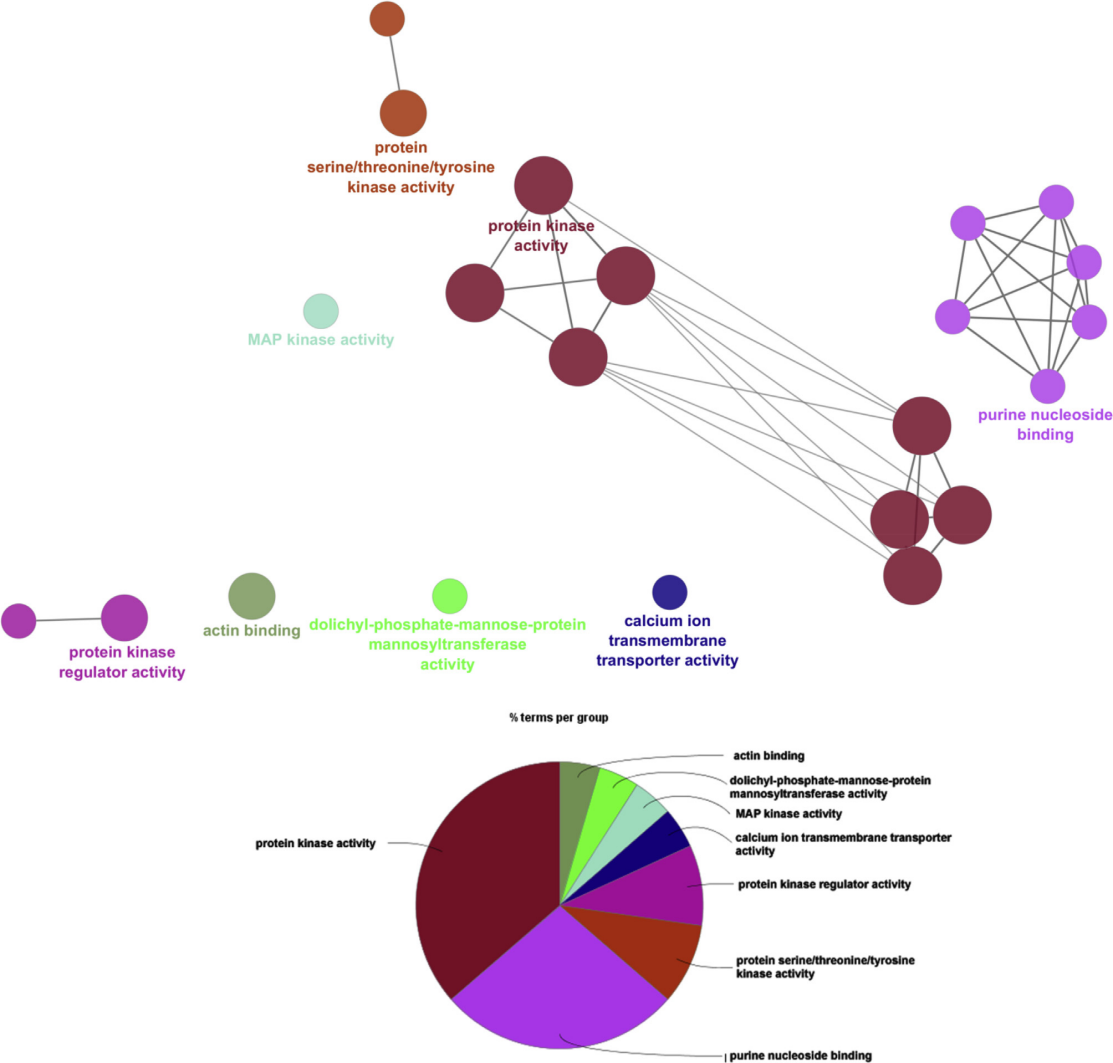
ClueGO analysis of top scoring clusters from molecular function point.

Based on molecular function ontology, annotations of these proteins were classified into major molecular functions such as protein kinase activity (GO:0004672), purine nucleoside binding (GO:0001883), protein serine/threonine/tyrosine kinase activity (GO:0004712), protein kinase regulator activity (GO:0019887), calcium ion transmembrane transporter activity (GO:0015085), MAP kinase activity (GO:0004707), dolichyl-phosphate-mannose-protein mannosyltransferase activity (GO:0004169) and actin binding (GO:0003779) (Fig. 6 and Table S2).

**Fig. 7:**
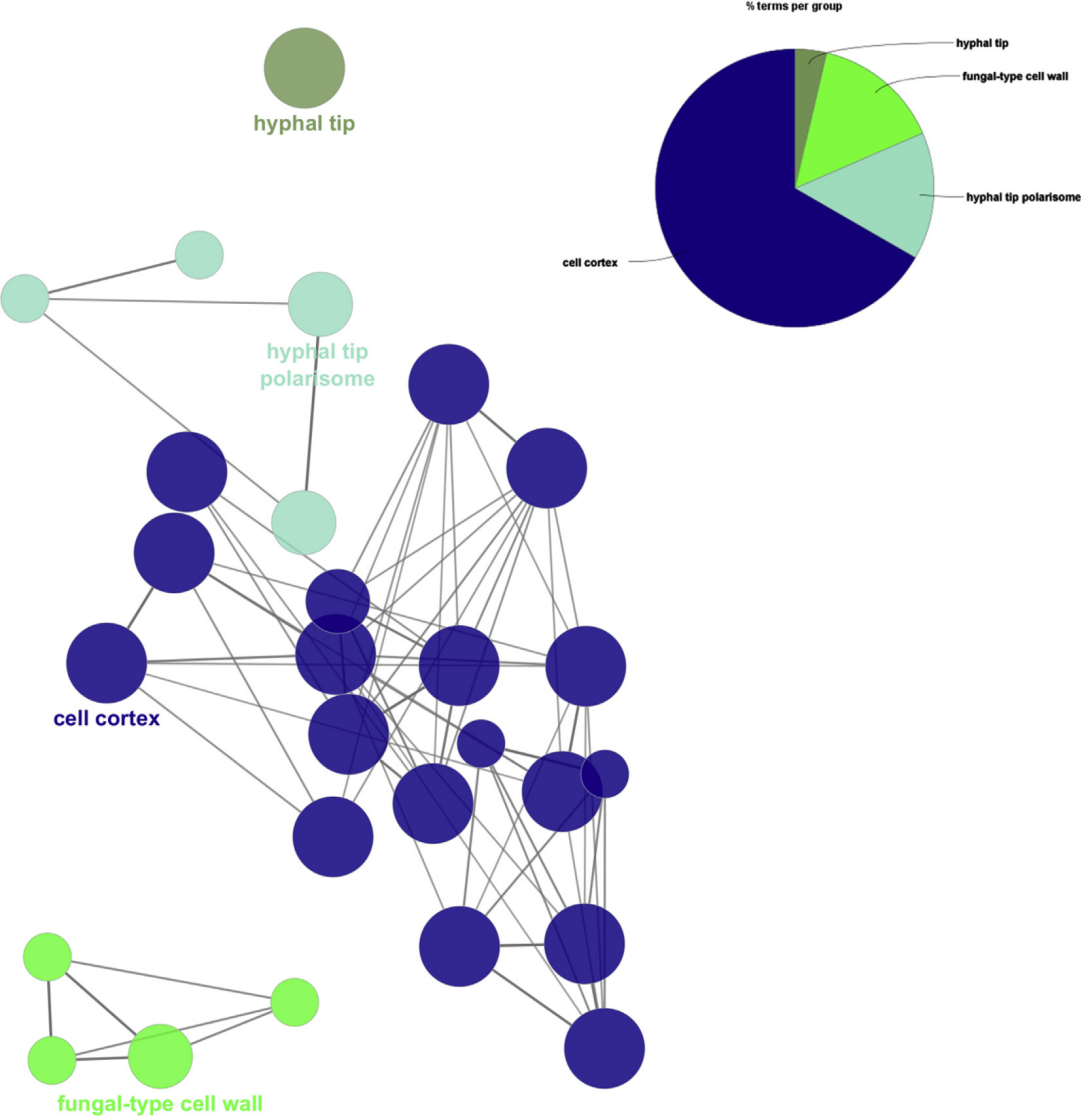
ClueGO analysis of top scoring clusters of cellular components.

Similarly, the major cellular components were functionally categorized into cell cortex (GO:0005938), hyphal tip (GO:0001411), fungal-type cell wall (GO:0009277) (Fig. 7 and Table S3).

### 3.6 Enrichment of proteins solely present in C. albicans and morphology analysis

**Fig. 8:**
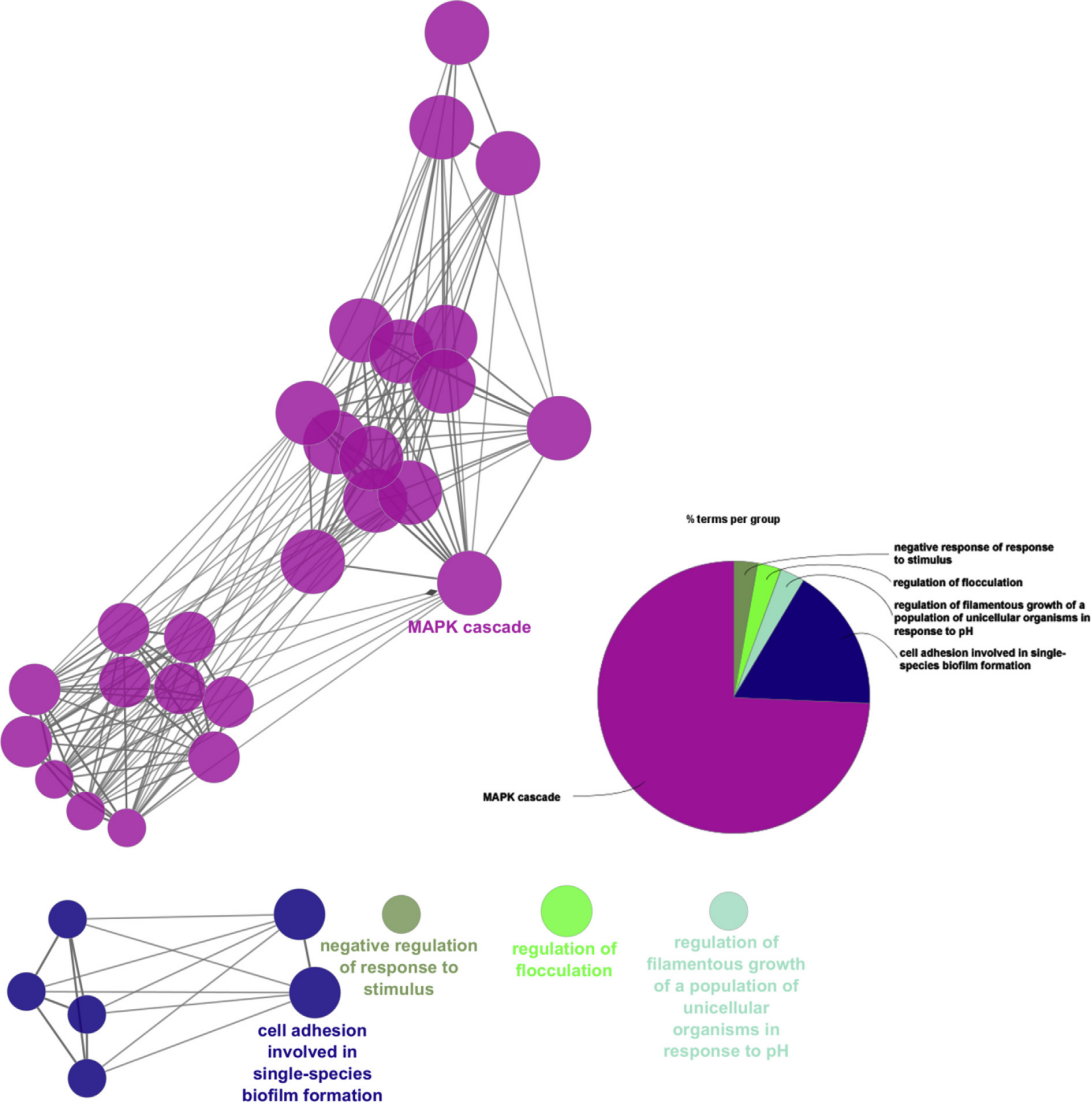
ClueGO analysis of unique genes from *C. albicans* in different biological process.

**Fig. 9:**
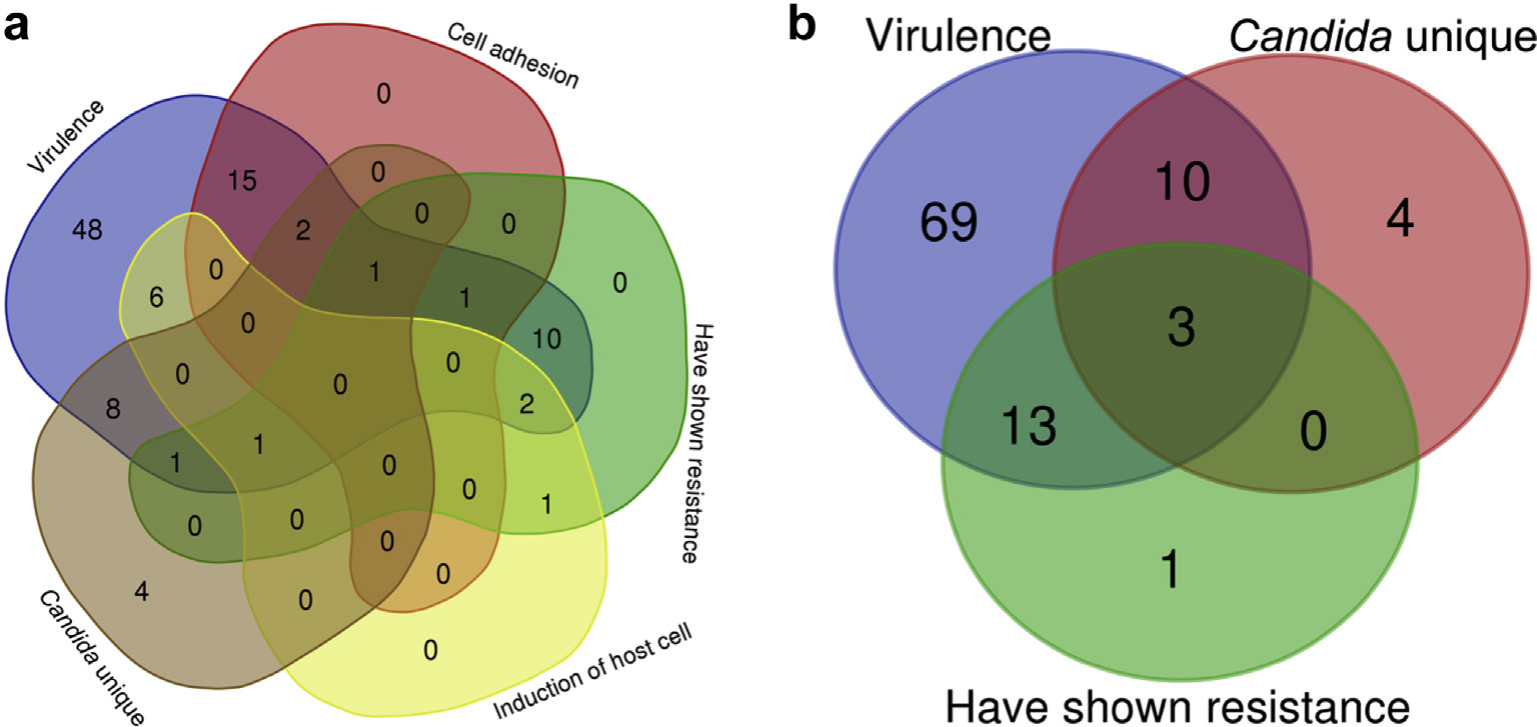
Venn diagram of proteins involved in different morphological features; (a) Categorization of proteins into virulence, cell adhesion, host resistant protein, induction to host, and proteins that are unique in *C. albicans*. (b) Small categorization of virulence, showed resistances and that were unique in *C. albicans*.

We prepared a list of 17 proteins that were unique in *C. albicans* and were not present in any yeast family. Functional annotations of their biological processes suggested that they can be grouped in only five categories such as MAPK cascade (GO:0000165), adhesion involved in single-species biofilm formation (GO:0043709) cell adhesion involved in single-species biofilm formation (GO:0043709), negative regulation of response to stimulus (GO:0048585), and regulation of filamentous growth of a population of unicellular organisms in response to pH (GO:1900741) (Fig. 8). From our dataset, 95 proteins of *C. albicans* have the potentiality to contribute towards virulence, 19 belong to cell adhesion group, 17 showed resistances to drugs/chemicals and 10 were found to be involved in host cell induction. By a comparative study, we observed that Bcr1 was a unique virulence protein in *C. albicans* which involved in symbiotic interaction and has roles in biofilm formation. Similarly, the Mkc1 was also a unique virulence protein which has shown resistance and participates in induction of host cell. The details were presented in Fig. 9 and Table S4.

### 3.7 Characterization of crucial proteins

**Fig. 10:**
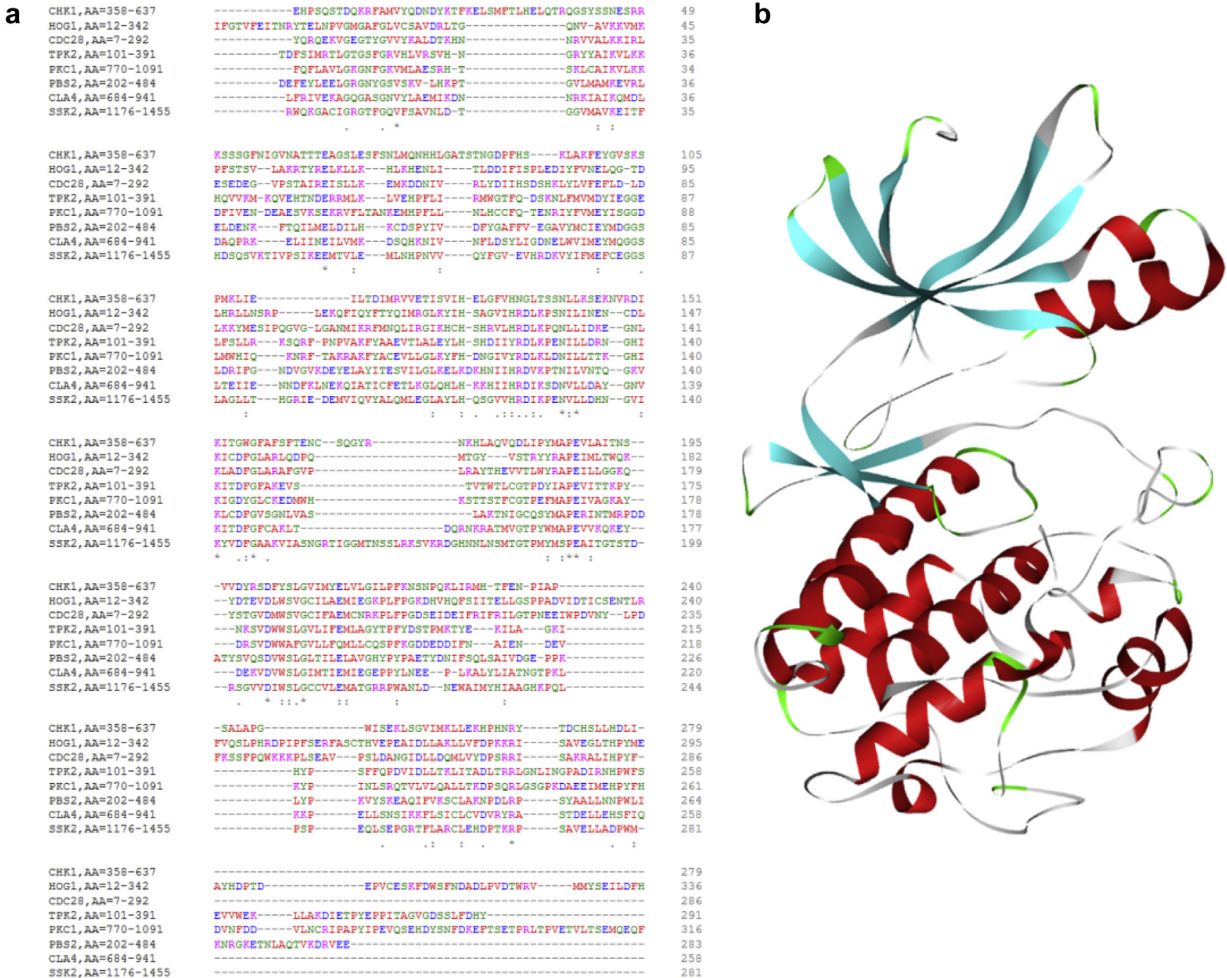
Characterization of kinase proteins in hyphae formation; (a) Sequence alignment of kinase domains, (b) Structural representation of modeled Chk1 kinase domain.

The top 20% of nodes having higher degrees, functional enrichment analysis and multiple morphological features reveals that the proteins with kinase activity were predominant. Among them Hog1, Ssk2, Pbs2, Chk1, Cdc28, Tpk2, Pkc1& Cla4 were the leading kinases showing variable roles in candida hyphae formation. Sequence alignment of this large family of kinase proteins showed <30% of sequence similarity among themselves (Fig. 10a). Additionally many of them are already declared as drug target due to their virulence property in candidiasis [57, 58, 59, 60, 61, 62]. Among them, a histidine kinase protein Chk1 that promotes hyphae formation, has been least studied. It is a large multifunctional protein of 2,471 amino acid lengths, an essential virulent protein in *Candida*, and a non-human homolog. structure of *Candida* Chk1 kinase domain is shown in Fig. 10b.

## 4 Discussion

Recent studies have revealed the pathological importance of *C. albicans* through its hyphae formation. The proteins responsible for hyphae formation are considered as the integral components for the major virulence strategy of *C. albicans*. Expressions and the interactions of these proteins are believed to exert various cellular functions, adaptation to adverse conditions, and inducing pathogenesis. Network analysis in the article is an informative tool to direct novel experimentation to provide further insight into the mechanism of pathogenesis and virulence of *C. albicans*. Hence, the understanding of these PPIs is essential to study the pathogenic mechanisms in *C. albicans* and also for developing new therapeutic strategies. In this work, we constructed a network of 137 proteins that have role in hyphae development and studied their functions by network topology, hub, clustering, and functional enrichment analysis.

Topological analysis confirmed our network as biologically scale-free and robust. From the average clustering coefficient and number of shortest path values, it was ascertained that the connectivity among proteins were very high. Centrality analysis of our predicted hyphae network yielded information regarding hubs which further helped to identify the hub proteins using CytoHubba. Out of the total 137 proteins, 18 possesses more than 20° among which Act1, Cdc28, Cdc42, Cla4 & Cyr1 were identified as interactome with the largest connections and were also involved in the promotion of hyphal growth, whereas Hog1, Hsp90, Pbs2, Pkc1 &Tpk1 were recognized as major suppressor of hyphal growth with the largest number of connections.

Modular analysis by MCODE produce three large clusters of proteins that were highly connected in the network based on their functional properties. Ontology and functional enrichment analyses of these clusters revealed that the proteins in these clusters were represented in groups such as nucleotide-binding, kinase activity, GTPase activity, filamentous growth, MAPK signaling, and other signaling pathways. Twelve out of the 18 identified hub proteins were reestablished within these clusters suggesting that these proteins were the key players in hyphae development in *C. albicans*.

Functional annotation analyzed by ClueGO provides a broad classification of these proteins and their involvement in various biological activities in addition to hyphal growth. Similarly, the ClueGO predicted three large clusters of proteins categorized on the basis of molecular function, biological process and cellular components. Within the molecular function, protein kinase activity is the largest one having 21 numbers of nodes and also kinase regulatory activity showed six nodes. Hence, the pathways related to kinase activity can be considered as one of the most important paths in *Candida* hyphae formation. Additionally, most of the proteins from the network were predicted to be localized on cell cortex or periphery of the fungi, which means they would act as the main protagonist in inducing infections within the host. Regulation of filamentous growth is the biological process that covered maximum number of protein nodes.

Cyr1 or Cdc35 is an essential enzyme of *C. albicans* that is associated in integrating the environmental signals from a range of sources responsible for hyphae formation. Induction of hyphae is further transmitted through interaction of Cyr1 with Ras1 and Cap1 [63]. This study postulated Cyr1 as one of the hub proteins having 38 connections. Cyr1 and its interacting partners were predicted to be involved in biological processes such as interaction with host, cellular response to oxygen containing compound and regulation of response to stress. Additionally, Cyr1 was found to bear the highest numbers of biological activities from the list of proteins considered.

Act1 is a hyphal tip associated protein and is required for hyphae elongation through hyphal tip polarization [64]. It also helps in localizing the Cdc42 during the hyphal development. Cdc42 plays the role of master regulator of polarity control and is known to interact with many PAK family kinases during the filament growth [65]. It is also proved that the Cdc28 controls the activities of Cdc42 and other hyphae associated proteins. Hence, the repression or inhibition of Cdc28 can disrupt the hyphal formation in *Candida*. The interactomes of these above proteins were predicted to be involved in biological processes such as mitotic cell cycle process, cell morphogenesis, regulation of filamentous growth, intracellular signal transduction, and regulation of cellular component organization. Act1 was found to interact with many hyphae regulating proteins.

In our network, Hog1 and Hsp90 possessed the maximum number of connections and were believed to be hyphae suppressing proteins in *C. albicans*. Lowering the Hog1 basal activity can promote Brg1 expression for hyphal elongation [66]. Similarly, Hsp90 regulates hyphal development by regulating Cyr1 and repressing Ras1-PKA signaling [67]. Hog1 was predicted with 11 biological processes and is involved in many regulatory pathways including regulation of filamentous growth and regulation of response to extracellular response. Both Hog1 and Hsp90 were found to form many genetic and physical interactions with the nodes that are involved in either hyphae development or suppression. Among the other hub proteins from hyphae suppressing group Pbs2, Pkc1, Tpk2 and Cdc24 were mainly associated with the others for their activation or inactivation purposes. The interacting partners of these proteins were predicted to be involved in processes like signal transduction and regulation of response to stimulus.

Among the hyphae forming proteins showing kinase activity, Hog1, Cek1 & Mkc1 proteins are the most essential kinases. In favorable condition hyphae formation takes place through Cek1 pathway. The activated Cek1 participates in the morphological transition through Cph1 hyphae specific transcription factor. The Cek1 protein is dephosphorylated by the phosphatase protein Cpp1. Cpp1 in turn is activated by Hog1 [68]. Thus Hog1 pathway suppresses the Cek1 activity and restrains the hyphae formation. The activated Hog1 protein also phosphorylate Mkc1 pathway which in turn promote cell wall integrity. Mkc1 is also stimulated by upstream protein Pkc1 [69]. Through this study Hog1 stress adaptation kinase pathway core components Ssk2, Pbs2 and Hog1 were found to be the most important hub hyphae proteins which are deduced in this study. Hog1 belongs to the MAP kinase family protein known for suppressing hyphae formation. It is a virulent and essential protein in *Candida*. Though its human homolog is present, it is considered as a drug target [57]. Pbs2 belongs to the MAP kinase family protein suppressing hyphae formation. It is a nonessential protein having human homolog [70]. Ssk2 and Pkc1 are also MAP kinase family proteins which suppress hyphae formation, and are already reported as drug target [57, 61]. Tpk2 belongs to the cAMP-PKA kinase family protein that is nonessential and are known to suppress the hyphae formation, but reported as drug target [71]. Cdc28 is an essential cyclin dependent kinase family protein in *Candida* that promotes the hyphae formation [72]. Chk1 is a histidine kinase protein that plays a crucial role in the yeast to hyphae transformation, biofilm formation, virulence, quorum sensing, peroxide adaptation, cell wall composition and triazole resistance [73, 74, 75, 76, 77, 78, 79, 80]. During *C. albicans* infection Chk1 is needed for the survival in neutrophils and adherence to esophageal cells in human [81]. From the above stated functions of the hub kinase protein Chk1, it is found to be an essential protein, different from its human homolog and we propose to consider it as a therapeutic or drug target for candidiasis involving hyphae formation. The findings on kinase pathways and the presence of predominant kinase hub proteins involved in hyphae formation make them suitable candidates which can be considered as potential targets for prevention of hyphae formation as well as for the development of new antifungal strategies.

The proteins that showed multiple morphological features were Bcr1, Mkc1, Hwp1, Als1, Pmr1, Sod5, Cek1 and Pmt2. Most of them had direct interactions with the hyphae suppressing proteins such as Hog1, Hsp90 and Nrg1. Among these, Pmr1 is the most explored one, which shares 13 connections in the network, having positive role in hyphae formation and development. The other proteins require further attention to understand their role in hyphae formation in *C. albicans*. It is also to be noted that the hyphae formation is induced under different cellular conditions. However, in this case we considered only those data which were verified by wet-lab experiments. The data used in our study were generated considering the cellular conditions. Since the proposed model in this work was based on the experimentally verified data, the model took into account the different cellular conditions inherently.

Overall, this study emphasizes on the involvement of major hyphae forming proteins in different cellular physiology of *C. albicans*, and their respective interactions necessary for the pathophysiology. From the study, it was concluded that the metabolic processes such as cAMP-mediated signaling, MAPK pathway and protein kinase pathway are significant for the morphogenesis and pathological activity of *C. albicans*. Proteins that are involved in both positive and negative regulation of hyphae formation are explored through network analyses and have also been described for their potential therapeutic targets. Further studies are in progress to elucidate the mechanisms for regulation of hyphae formation in *C. albicans*.

## Declarations

### Author contribution statement

Sanjib Das, Rajabrata Bhuyan, Angshuman Bagchi, Tanima Saha: Conceived and designed the experiments; Performed the experiments; Analyzed and interpreted the data; Contributed reagents, materials, analysis tools or data; Wrote the paper.

### Funding statement

This work was supported by grants from BTIS net programme of DBT, Ministry of Science and Technology, Government of India, New Delhi. Angshuman Bagchi and Tanima Saha also received financial assistance from Personal Research Grant 2018–2019 provided by University of Kalyani.

### Competing interest statement

The authors declare no conflict of interest.

### Additional information

No additional information is available for this paper.

## Appendix A Supplementary data

Revised_Supplementary_Material Revised_Supplementary_Material
