# Supplementary material for: Network analysis of hyphae forming proteins in Candida albicans identifies important proteins responsible for pathovirulence in the organism
Source: Heliyon. 2019 Jun 13;5(6):e01916. doi: 10.1016/j.heliyon.2019.e01916 (PMC6580234; doi:10.1016/j.heliyon.2019.e01916)
Supplement: Revised_Supplementary_Material [file mmc1.docx]

| **Table S1. Functional enrichment of biological process** | | | | |
| --- | --- | --- | --- | --- |
| **Biological process** | **GO ID** | ***P* Value** | ***P*Value corrected** | **Proteins involved** |
| cell morphogenesis | GO:0000902 | 3.30E-07 | 6.14E-05 | Bem1, Cbk1, Cdc24, Cdc42, Myo2, Pde2, Sho1, Tpk1, Tpk2 |
| cell wall organization | GO:0071555 | 2.46E-15 | 2.46E-15 | Cbk1, Cdc10, Cek1, Chk1, Cmp1, Cph1, Cst20, Hog1, Hst7, Hwp1, Hxk1, Hym1, Mid1, Mkc1, Mob2, Pbs2, Pde2, Pkc1, Pmr1, Pmt1, Rbt1, Rhb1, Rvs167, Sho1, Sln1, Ssk1, Tpk1 |
| cellular bud site selection | GO:0000282 | 7.31E-08 | 1.40E-05 | Bni1, Bud2, Crz1, Int1, Rax2, Rsr1, Spa2 |
| cellular response to abiotic stimulus | GO:0071214 | 3.43E-21 | 8.44E-19 | Als1, Bud2, Cbk1, Cch1, Ccn1, Cln3, Cmp1, Crk1, Crz1, Gcn4, Gis2, Gpa2, Hgt12, Hog1, Hym1, Mid1, Mns1, Mob2, Nrg1, Pbs2, Pmr1, Pmt6, Rim101, Rsr1, Sho1, Sln1, Ssk1, Ssk2, Ume6, Yak1, Ypd1 |
| cellular response to drug | GO:0035690 | 2.44E-10 | 5.21E-08 | Cbk1, Ccn1, Cdr1, Cek1, Cla4, Clb2, Cln3, Cmp1, Crz1, Cyr1, Gis2, Hsl1, Hsp90, Hst7, Hym1, Mig1, Mkc1, Mob2, Ndt80, Nrg1, Pde2, Pkc1, Pmt1, Pmt2, Pmt6, Rbt4, Rhb1, Rim101, Ssn6, Tpk1, Tup1, Yck2 |
| cellular response to mechanical stimulus | GO:0071260 | 1.25E-07 | 2.40E-05 | Cch1, Crz1, Mid1, Mns1, Pmr1 |
| cellular response to organic substance | GO:0071310 | 2.15E-12 | 4.83E-10 | Bud5, Cdr1, Chk1, Cph1, Gcn4, Gpa2, Gpr1, Hgt12, Hsp90, Hst7, Ndt80, Nrg1, Sln1, Tec1, Tup1 |
| cellular response to oxidative stress | GO:0034599 | 3.44E-07 | 6.37E-05 | Cdr1, Ddr48, Ecm7, Gal10, Gre2, Hog1, Mkc1, Ndt80, Sac6, Sho1, Sod5, Ssk1, Ssk2 |
| cellular response to oxygen-containing compound | GO:1901701 | 6.03E-11 | 1.30E-08 | Bud5, Chk1, Cyr1, Gcn4, Gpa2, Gpr1, Hgt12, Mkc1, Ndt80, Nrg1, Pde2, Sln1, Sod5, Tup1 |
| chlamydospore formation | GO:0001410 | 2.74E-07 | 5.19E-05 | Cdc10, Cdc11, Cla4, Cst20, Hog1, Nrg1, Rim101, Sch9 |
| cytoskeleton organization | GO:0007010 | 1.07E-10 | 2.30E-08 | Act1, Bni1, Bud2, Bud6, Cdc10, Cdc11, Cdc12, Cdc14, Dbf2, Dyn1, Mob2, Myo2, Pfy1, Sla1, Sla2, Spa2 |
| development involved in symbiotic interaction | GO:0044111 | 3.45E-07 | 6.34E-05 | Cla4, Cln3, Cph1, Hxk1, Nrg1, Sch9, Tpk2, Tup1 |
| filamentous growth of a population of unicellular organisms in response to chemical stimulus | GO:0036171 | 1.33E-08 | 2.68E-06 | Bni1, Cyr1, Gcn4, Gpr1, Ndt80, Nrg1, Rad53, Rhb1, Rim101, Sch9, Tpk1, Tup1 |
| fungal-type cell wall biogenesis | GO:0009272 | 2.28E-05 | 0.003668754 | Cek1, Chk1, Gal10, Mns1, Pkc1, Sac1, Sho1, Vam3 |
| growth involved in symbiotic interaction | GO:0044110 | 1.78E-06 | 3.09E-04 | Bcr1, Hsl1, Hwp1, Hxk1, Int1, Pmt1, Rgt1 |
| growth of unicellular organism as a thread of attached cells | GO:0070783 | 2.27E-08 | 4.52E-06 | Cdc10, Cdc11, Cdc14, Cdc24, Cdc42, Cdc53, Cph1, Cyr1, Gal10, Gpa2, Hal9, Hsl1, Myo2 |
| import into cell | GO:0098657 | 1.67E-05 | 0.002707995 | Chk1, Mid1, Rbt5, Rvs167, Sla1, Sla2, Vam3 |
| interaction with host | GO:0051701 | 8.22E-06 | 0.001380587 | Als1, Cph1, Cyr1, Gpr1, Rim101, Sod5, Ssk1, Tpk2, Tup1, Vps51, Yck2, Yhb1 |
| intracellular signal transduction | GO:0035556 | 9.18E-20 | 2.25E-17 | Bem3, Bud2, Bud5, Cdc24, Cdc42, Cek1, Chk1, Cpp1, Crz1, Cst20, Cyr1, Gpa2, Hog1, Hst7, Mkc1, Pbs2, Pde2, Pho84, Pkc1, Ptp3, Rga2, Rho3, Rsr1, Sln1, Ssk1, Ssk2, Ypd1 |
| mitotic cell cycle process | GO:1903047 | 6.32E-08 | 1.22E-05 | Bni1, Bud2, Ccn1, Cdc14, Cdc28, Cln3, Crz1, Dbf2, Dyn1, Int1, Rax2, Rsr1, Sla2, Spa2 |
| negative regulation of filamentous growth of a population of unicellular organisms | GO:1900429 | 2.40E-13 | 5.53E-11 | Bcy1, Cln3, Cpp1, Crk1, Gal10, Gpr1, Hal9, Hog1, Hsl1, Hsp90, Nrg1, Pde2, Rgt1, Sch9, Ssn6, Tpk1, Tup1, Tye7 |
| negative regulation of response to stimulus | GO:0048585 | 1.81E-09 | 3.74E-07 | Bcy1, Cpp1, Crk1, Gpr1, Hog1, Nrg1, Pde2, Ptp3, Sch9, Tpk1 |
| nuclear migration | GO:0007097 | 1.48E-05 | 0.002430387 | Cdc10, Cdc11, Dyn1, Myo2 |
| positive regulation of response to external stimulus | GO:0032103 | 6.31E-12 | 1.39E-09 | Cek1, Cla4, Cph1, Crz1, Cst20, Cyr1, Gpa2, Hst7, Mkc1, Sch9, Ssk1, Tpk2, Ume6 |
| regulation of cellular component organization | GO:0051128 | 8.14E-05 | 0.012047079 | Bud6, Cdc14, Cdc42, Chk1, Cst20, Dbf2, Hsl1, Mkc1, Pde2 |
| regulation of filamentous growth | GO:0010570 | 1.82E-33 | 4.67E-31 | Bcy1, Cbk1, Ccn1, Cdc24, Cdc42, Cdc53, Cek1, Chk1, Cla4, Cln3, Cph1, Cpp1, Crk1, Crz1, Cst20, Cyr1, Czf1, Gal10, Gcn4, Gpa2, Gpr1, Hal9, Hog1, Hsl1, Hsp90, Hst7, Mkc1, Mob2, Myo2, Ndt80, Nrg1, Pde2, Rad53, Rgt1, Rim101, Sch9, Sho1, Sln1, Ssk1, Ssn6, Tec1, Tpk1, Tpk2, Tup1, Tye7, Ume6, Yck2 |
| regulation of filamentous growth of a population of unicellular organisms | GO:1900428 | 1.34E-31 | 3.41E-29 | Bcy1, Cbk1, Ccn1, Cdc24, Cdc42, Cek1, Chk1, Cla4, Cln3, Cph1, Cpp1, Crk1, Crz1, Cst20, Cyr1, Czf1, Gal10, Gcn4, Gpa2, Gpr1, Hal9, Hog1, Hsl1, Hsp90, Hst7, Mkc1, Mob2, Myo2, Ndt80, Nrg1, Pde2, Rad53, Rgt1, Rim101, Sch9, Sho1, Sln1, Ssk1, Ssn6, Tec1, Tpk1, Tpk2, Tup1, Tye7, Ume6 |
| regulation of phosphorylation | GO:0042325 | 1.35E-09 | 1.35E-09 | Bcy1, Ccn1, Clb2, Clb4, Cln3, Cpp1, Hst7, Mob2, Pbs2, Ptp3, Ssk2, Tye7 |
| response to lipid | GO:0033993 | 6.04E-06 | 0.001026752 | Cdr1, Chk1, Hsp90, Sln1, Tup1 |
| signal transduction | GO:0007165 | 2.95E-24 | 7.49E-22 | Bem3, Bud2, Bud5, Cdc24, Cdc42, Cek1, Chk1, Cpp1, Crz1, Cst20, Cyr1, Gpa2, Gpr1, Hgt12, Hog1, Hsp90, Hst7, Mep2, Mkc1, Pbs2, Pde2, Pho84, Pkc1, Ptp3, Ras1, Rga2, Rhb1, Rho3, Rim101, Rsr1, Sho1, Sln1, Ssk1, Ssk2, Ypd1 |
| signal transduction by protein phosphorylation | GO:0023014 | 7.38E-12 | 1.62E-09 | Cek1, Chk1, Cpp1, Cst20, Hog1, Hst7, Mkc1, Pbs2, Ptp3, Sln1, Ssk2 |
| single-species biofilm formation | GO:0044010 | 3.51E-14 | 8.22E-12 | Als1, Bcr1, Cbk1, Chk1, Cln3, Cph1, Cyr1, Czf1, Ece1, Gcn4, Hwp1, Mkc1, Mob2, Ndt80, Pde2, Pmt1, Pmt2, Pmt6, Rbt5, Rhb1, Tec1, Tpk1, Tpk2, Tye7, Vam3, Yak1, Yck2 |

| **Table S2. Functional enrichment on the basis of molecular functions** | | | | |
| --- | --- | --- | --- | --- |
| **Molecular functions** | **GO ID** | ***P* Value** | ***P* Value corrected** | **Proteins involved** |
| MAP kinase activity | GO:0004707 | 2.78E-04 | 0.008332411 | Cek1, Hog1, Mkc1 |
| actin binding | GO:0003779 | 8.14E-05 | 0.002766077 | Bni1, Myo2, Pfy1, Sac6, Sla1, Sla2 |
| calcium ion transmembrane transporter activity | GO:0015085 | 0.001036354 | 0.026945209 | Cch1, Mid1, Pmr1 |
| dolichyl-phosphate-mannose-protein mannosyltransferase activity | GO:0004169 | 4.11E-04 | 0.011927191 | Pmt1, Pmt2, Pmt6 |
| protein kinase activity | GO:0004672 | 9.51E-13 | 4.09E-11 | Cbk1, Cdc28, Cek1, Chk1, Cla4, Crk1, Cst20, Dbf2, Hog1, Hsl1, Hst7, Mkc1, Pbs2, Pkc1, Rad53, Sch9, Sln1, Ssk2, Tpk2, Yak1, Yck2 |
| protein kinase regulator activity | GO:0019887 | 2.34E-05 | 8.19E-04 | Bcy1, Ccn1, Clb2, Clb4, Cln3, Mob2 |
| protein serine/threonine/tyrosine kinase activity | GO:0004712 | 1.02E-04 | 0.003359073 | Hst7, Pbs2, Rad53 |
| purine nucleoside binding | GO:0001883 | 1.63E-04 | 0.005201215 | Cdc10, Cdc11, Cdc12, Cdc42, Gpa2, Ras1, Rhb1, Rho3, Rsr1, Sep7 |

| **Table S3. Functional enrichment of cellular components** | | | | |
| --- | --- | --- | --- | --- |
| **Cellular components** | **GO ID** | ***P* Value** | ***P* Value corrected** | **Proteins involved** |
| cell cortex | GO:0005938 | 1.47E-18 | 4.10E-17 | Act1, Bni1, Bud2, Bud6, Cbk1, Cdc10, Cdc11, Cdc12, Cdc42, Hsl1, Int1, Mob2, Rga2, Rsr1, Rvs167, Sac6, Sep7, Sla1, Sla2, Smt3, Spa2 |
| fungal-type cell wall | GO:0009277 | 3.59E-04 | 0.004667 | Als1, Bud2, Cht2, Ddr48, Ece1, Gap1, Hsp12, Hsp90, Hwp1, Rbt1, Rbt5, Rsr1, Sod5 |
| hyphal tip | GO:0001411 | 2.34E-13 | 5.84E-12 | Act1, Bem3, Bni1, Bud2, Bud5, Bud6, Cbk1, Cdc10, Cdc11, Cdc24, Cdc42, Mob2, Rax2, Sec2, Spa2 |
| hyphal tip polarisome | GO:0031562 | 1.06E-04 | 0.001593 | Bud6, Cdc42, Spa2 |

| **Table S4. Proteins that participate in multiple morphological features** | | | | | | | | | | | | |
| --- | --- | --- | --- | --- | --- | --- | --- | --- | --- | --- | --- | --- |
| **Candida Unique \| Cell adhesion \| Have shown resistance \| Virulence** | **Candida Unique \| Have shown resistance\| Induction of host cell \| Virulence** | **Candida Unique \| Cell adhesion\| Virulence** | **Have shown resistance\| Induction of host cell\| Virulence** | **Candida Unique \| Have shown resistance\| Virulence** | **Candida unique \| Have shown resistance \| Virulence** | **Have shown resistance \| Induction of host cell** | **Cell adhesion\| Virulence** | **Induction of host cell \| Virulence** | **Candida Unique \| Virulence** | **Have shown resistance \| Induction of host cell** | **Candida unique \| Virulence** | **Have shown resistance \| Virulence** |
| Bcr1 | Mkc1 | Hwp1 Als1 | Pmr1 Sod5 | Cek1 | Mkc1 Bcr1 Cek1 | Pmt2 | Pmt1 Czf1 Int1 Csp37 Tup1 Pde2 Phr1 Cdc10 Rhr2 Hsl1 Pmt6 Sac1 Tec1 Rvs167 Gap1 | Phr2 Och1 Rhd3 Rim101 | Rbt4 Cpp1 Ece1 Crk1 Rbt1 Hst7 Rbt5 Cst20 | Rbt4 Cpp1 Ece1 Crk1 Rbt1 Hst7 Rbt5 Cst20 | Rbt4 Cpp1 Ece1 Crk1 Rbt1 Hst7 Rbt5 Hwp1 Cst20 Als1 | Yhb1 Ssk1 Chk1 Cyr1 Hog1 Cph1 Ras1 Pmr1 Sod5 Mns1 Cla4 |
